# Supplementary material for: A Specific HPLC Method to Determine Residual HEPES in [68Ga]Ga-Radiopharmaceuticals: Development and Validation
Source: Molecules. 2022 Jul 13;27(14):4477. doi: 10.3390/molecules27144477 (PMC9323806; doi:10.3390/molecules27144477)
Supplement: Supplementary file 1 [file molecules-27-04477-s001.zip › molecules-1790144-supplementary-revised.pdf]

Supplementary Materials

Table S1. Precision and accuracy of the calibration curves for HEPES (five replicates of eight standard in the expected range of concentration values).

Reference value of concentration of HEPES [µg/mL]	Calculated concentration [µg/mL]	Calculated average concentration [µg/mL]	DS	CV%	Average Bias%
100	100.65<br>100.69<br>100.67<br>100.74<br>100.71	100.69	0.04	0.03	100.69
80	78.80<br>80.03<br>78.44<br>79.48<br>78.97	79.14	0.62	0.78	98.93
60	60.68<br>60.44<br>61.13<br>60.71<br>60.79	60.75	0.25	0.41	101.25
40	37.80<br>37.97<br>38.14<br>38.20<br>38.08	38.04	0.16	0.42	95.10
20	20.62<br>21.34<br>20.37<br>20.48<br>20.82	20.82	0.33	1.57	104.12
10	10.27<br>10.30<br>10.65<br>10.62<br>10.67	10.50	0.20	1.90	104.98
5	4.82<br>4.78<br>4.83<br>5.01<br>4.85	4.86	0.09	1.83	97.15
3	2.97<br>3.12<br>3.05<br>3.02<br>3.02	3.04	0.06	1.84	101.17

Table S2. Accuracy and intraday precision for the HPLC method used for HEPES (n = 5).

	Reference value of concentration of HEPES [µg/mL]	Average concentration [µg/mL, means ± SD]	Accuracy (Bias %)	Precision (CV%)
Day 1	90	88.58 ± 0.15	98.42	0.17
Day 2		88.63 ± 0.08	98.47	0.09
Day 3		88.61 ± 0.13	98.46	0.15

Day 1		49.10 ± 0.67	98.20	1.37
Day 2	50	49.11 ± 0.65	98.21	1.33
Day 3		49.15 ± 0.04	98.29	0.09
Day 1		3.89 ± 0.18	97.14	1.78
Day 2	4	3.88 ± 0.15	97.08	1.93
Day 3		3.87 ±0.14	96.73	1.99

Table S3. Accuracy and inter-day precision for the HPLC method used for HEPES (*n* = 15).

	**Reference value of concentration of Ga-NODAGA-exendin-4 [µg/mL]**	**Average concentration [µg/mL]**	**Accuracy (Bias %)**	**Precision (CV%)**
Day 1		4.70 ± 0.006	4.50	0.12
Day 2	4,50	4.71 ± 0.004	4.59	0.09
Day 3		4.71 ± 0.006	4.58	0.12
Day 1		2.02 ± 0.002	1.08	0,10
Day 2	2,00	2.02 ± 0.002	1.14	0.08
Day 3		2.01 ± 0.001	0.70	0.07
Day 1		0.81 ± 0.001	-4.34	0.09
Day 2	0,85	0.81 ± 0.001	-4.43	0.09
Day 3		0.81 ± 0.001	-4.75	0.07
